# Relationship Between the Neutrophil to Lymphocyte Ratio and the Presence and Size of Thyroid Nodules

**DOI:** 10.7759/cureus.3866

**Published:** 2019-01-11

**Authors:** Elcin Erdogan Yucel, Sibel Demiral Sezer

**Affiliations:** 1 Internal Medicine, University of Health Sciences Tepecik Training and Research Hospital, Izmir, TUR

**Keywords:** thyroid nodule, neutrophil-to-lymphocyte ratio, inflammation, mean platelet volume

## Abstract

Aim

To investigate the possible relationship between the neutrophil to lymphocyte ratio (NLR) value and the presence and size of thyroid nodules.

Patients and methods

Data of 216 patients between 18 and 65 years of age, who had presented to the internal medicine department of our hospital between February 2018 and September 2018, were collected retrospectively. A total of 216 patients fulfilling the inclusion criteria were included in the study.

Results

An ultrasonographic examination revealed thyroid nodules in 105 patients and no thyroid nodules in 111 patients. Among those with a nodule, 52 had a nodule size of ≤10 mm and 53 had a nodule size of >10 mm. These groups were compared with regard to age, gender, body mass index, thyroid function tests, neutrophil, lymphocyte and platelet count, NLR, and mean platelet volume (MPV). Although the absolute counts of lymphocytes and neutrophils were lower in the group with nodules, no significant difference was observed between the groups. Among those with a nodule, no significant difference was determined between nodule size and thyroid function tests, NLR, or MPV. In the group of patients with a nodule, malignant pathology was reported in 2.7%.

Conclusions

Although there are studies suggesting a relationship between NLR, MPV, and the presence of malignancy and mortality, we determined no such relationship between nodule formation and their size in thyroid tissue.

## Introduction

In areas of iodine deficiency, thyroid nodules have been detected via ultrasonographic screening in 19%-68% of the population. Although these nodules are usually of benign character, 7%-15% are malignant [1]. The systemic inflammatory response that develops against genetic and external factors has been demonstrated to play a role in the physiopathogenesis of malignant transformation [2].

Recently, the number of studies investigating the neutrophil to lymphocyte ratio (NLR) as an indicator of inflammatory response, which is an easy and cost-effective method for the purpose, has increased. A positive correlation has been demonstrated between the high NLR and the recurrence and mortality rates in pulmonary, gastric, colorectal, hepatocellular, and breast cancers [3-6].

Thyroid cancer is the most frequent endocrine malignancy. Studies have been published demonstrating a correlation between high NLR and increased mortality and poor prognosis in thyroid cancer [3,7]. The aim of this study was to investigate the possible relationship between the NLR value and the presence and size of thyroid nodules.

## Materials and methods

Patients and study design

The ultrasonographic data of 216 patients between the ages of 18 and 65 years, who presented to the outpatient clinics of the general internal diseases department of our hospital between February 2018 and September 2018 and fulfilled the inclusion criteria, were retrospectively investigated.

The inclusion criteria were: A history of thyroid dysfunction and/or previous thyroid inflammation detected via ultrasonography and admission to the general internal diseases department due to routine follow-up.

The exclusion criteria were: Diagnosed hematological/oncological diseases, coronary artery disease within the last six months, infectious disease or glucocorticoid use within the last three months, diabetes mellitus (DM), hypertension, connective tissue disorders, autoimmune diseases, renal or hepatic dysfunction, and smoking, which could change the serum white blood cell count and distribution.

Blood samples were collected from the antecubital vein between 08.00 and 10.00 am. Thyrotropin, free T4 (sT4), hemoglobin, neutrophil, lymphocyte, platelet, mean platelet volume (MPV), and C-reactive protein (CRP) values were analyzed using the Mindray BC-6800 (Shenzhen Mindray Biomedical Electronics, Nanshan, P.R. China) and the Beckman Coulter DX1800 immunoassay analyzer (Beckman Coulter Inc., CA, USA). The nodule size was calculated as the longest diameter measured ultrasonographically (Siemens SL-2; Siemens, Munich, Germany). Patients with a nodule size of ≥10 mm and/or microcalcification within the nodule underwent a fine needle aspiration biopsy. NLR was calculated by dividing the absolute neutrophil count by the absolute lymphocyte count. The patients were primarily investigated in two groups: those having or not having a nodule. Those with nodules were further grouped as nodule size ≤10 mm or >10 mm. The pathology reports of the fine needle aspiration biopsies of patients with a nodule size of ≥10 mm and/or microcalcification within the nodule were collected retrospectively.

These groups were compared with regard to age, gender, body mass index (BMI), thyroid function tests, neutrophil, lymphocyte, and platelet count, NLR, and MPV. Consent from the ethics committee was not required because of the retrospective nature of this study.

Statistical analysis

Numerical variables were expressed as mean and standard deviation. Categorical variables were expressed as numbers and percentages. The student's t-test was used for a comparison of the two groups for numerical variables, and the cross-table and chi-square tests were used for an analysis of the categorical variables. The data analysis was performed using Statistical Package for the Social Science (SPSS Inc, Chicago, Illinois, USA) version 22.0, and a p-value of <0.05 was considered significant.

## Results

We retrospectively reviewed the medical data of 305 patients. A total of 216 patients fulfilling the inclusion criteria were included in the study. The ultrasonographic examination revealed thyroid nodules in 105 patients and no thyroid nodules in 111 patients. Among those with a nodule (n=105), 52 had a nodule size of ≤10 mm and 53 had a nodule size of >10 mm.

No significant correlation was determined between body mass index (BMI), gender, thyrotropin, sT4, hemoglobin, leukocyte, neutrophil, and platelet count, NLR, or MPV. Although the absolute counts of lymphocytes and neutrophils were lower in the group with nodules, no significant difference was observed between the groups (Table 1). The presence and size of nodules were observed to increase with age.

**Table 1:** Comparison of demographic characteristics and biochemical parameters of patients with and without thyroid nodules Variables are expressed as mean ± standard deviation (SD) or (percentage). CRP, C-reactive protein; WBC, white blood cell; NLR, neutrophil to lymphocyte ratio; MPV, mean platelet volüme; BMI, body mass index

Variable	With nodules (n=105)	Without nodules (n=111)	p-value
Age (years)	44.8±12.7	35.9±11.8	<0.001
Sex: Male Female	14 (13.3%) 91 (86.7%)	16 (14.4%) 95 (85.6%)	0.818
BMI (kg/m^2^)	25.1± 1.4	26.2±1.3	0.912
Thyrotropin (mIU/L)	2.14±0.13	2.02±0.17	0.06
Free T4	0.93±0.31	0.89±0,028	0.317
CRP (mg/dl)	2.1±1.5	1.6±1.2	0.125
Hemoglobin (g/dl)	12.7±0.14	12.8±0.14	0.997
WBC count(/ul)	7560±197	8101± 189	0.049
Neutrophil (/ul)	4597±181	4860±159	0.269
Lymphocyte (/ul)	2282±80	2464±71	0.093
Platelet (10^9^/L)	266 ±6	269±6	0.718
NLR	2.22±0.113	2.10±0.86	0.414
MPV	8.77±0.97	8.90±0.95	0.174

Among those with a nodule, no significant difference was determined between nodule size and BMI, gender, thyroid function tests, hemoglobin, leukocyte, neutrophil, or platelet count, NLR, or MPV (Table 2). Among the 58 patients with a nodule size of ≥10 mm and microcalcifications determined via ultrasonography, a fine needle aspiration biopsy was performed on 56 (96.5%) patients who agreed to the procedure. Benign pathology was observed in 48 (85.7%), papillary carcinoma was detected in two (3.5%), and an oncocytic variant of follicular carcinoma was detected in one (1.7%). Suspicious but non-diagnostic cytology was observed in five (8.9%) patients. In the group of patients with a nodule, malignant pathology was reported in three (2.7%) patients.

**Table 2:** Comparison of demographic characteristics and biochemical parameters of patients with a nodule size of ≤10 mm and >10 mm Variables are expressed as mean ± standard deviation (SD) or (percentage). CRP, C-reactive protein; WBC, white blood cell; NLR, neutrophil to lymphocyte ratio; MPV, mean platelet volume

Variable	Nodule size ≤10 mm (n=52)	Nodule size >10 mm (n=53)	p-value
Age (years)	42±12.4	47.5±12.5	0.025
Sex: Male Female	3 (5.8%) 49 (94.2%)	11 (20.8%) 42 (79.2%)	0.024
Thyrotropin (mIU/L)	2.08±1.42	1.72±1.26	0.171
Free T4	0.90±0.24	0.96±0.38	0.355
CRP	1.8±1.1	2.3±0.9	0.745
Hemoglobin (g/dl)	12.6±1.47	13±1.46	0.120
WBC count (/ul)	7625±1967	7496±2086	0.746
Neutrophil (/ul)	2068±1152	2375±1160	0.176
Lymphocyte (/ul)	2334±680	2232±950	0.529
Platelet (/ul)	261596±49552	2232±950	0.458
NLR	2.06±1.15	2.37±1.16	0.176
MPV	8.8±1.04	8.7±0.96	0.660

## Discussion

A thyroid nodule is a frequently observed condition. It has been related to age, gender, smoking habit, obesity, alcohol intake, and metabolic syndrome [1,8-9]. The incidence of thyroid nodules has been demonstrated to increase in patients receiving radiotherapy to the head-neck region. Such clinical situations may be evaluated via a physical examination and anamnesis of the patient. In our study, we investigated the relationship between the NLR and CRP values and the presence and size of the nodules, the effects of inflammation on benign thyroid nodules, and whether these biochemical parameters could provide specialists with information about the presence and histology of the nodules or not.

Patients with cardiovascular diseases, malignancies, diabetes mellitus (DM), hypertension, and connective tissue diseases were excluded since studies have shown higher NLR values in such cases as compared to the healthy population [10-11].

The American Thyroid Association guide, published in 2015, reported that nodules were observed in 19%-68% of patients and more frequently in the female gender and in those of advanced age [1]. In our study, nodules were observed in 48.6% of the patients (n=105) and nodule prevalence and their sizes increased with an increase in age, which was in accordance with findings in the literature. Likewise, many studies have reported an increased prevalence of nodular goiter with age [1]. We detected no significant difference between gender and the presence of nodules. We believe that this was due to the fact that we had not designed the sample size for a prevalence study but had designed it for a prospective, sectional study that aimed to investigate the relationship between the groups. Studies including larger sample sizes will better reflect the relationship between the presence of nodules and gender.

Studies investigating the relationship between CRP and NLR are generally conducted on certain patient groups. Kocer et al. reported significantly elevated levels of NLR in papillary thyroid cancer, Kao et al. determined a positive correlation between CRP and NLR in malignant mesothelioma, and Ohno et al. detected this correlation in renal cell carcinoma [7,12-13]. In our study, however, we observed that CRP was not affected by the presence or size of a nodule. Likewise, no significant relationship was observed between the CRP and NLR values of patients. This was probably due to having excluded conditions that could cause high CRP levels such as inflammatory conditions or malignancies.

Studies have reported high NLR values, indicating increased mortality rates in colorectal, hepatocellular, gastric, and endocrine malignancies [4-6,11]. Liu et al. compared the outcomes in 159 patients with malignant thyroid nodules and 318 patients with benign nodules and reported a correlation between high preoperative NLR values and the tumor size and prognosis [14]. Many hypotheses have been proposed to explain this situation. Neutrophilia and/or lymphopenia have been accepted as indicators of increased NLR and inflammation. It has been demonstrated that the cytolytic activities of natural killer cells and lymphocytes have been inhibited with the increase in the neutrophil count and, therefore, neutrophil infiltration into tumor tissue has been accepted as an indicator of poor prognosis [15-16]. Inflammatory cytokines and chemokines, as well as neutrophils, are produced by the tumor cells. Neutrophils and other immune system cells secrete tumor growth-promoting factors, such as vascular endothelial growth factor, hepatocyte growth factor, interleukin 6, and interleukin 8, and the growth of tumor cells is stimulated. Therefore, increased NLR and interleukin levels are accompanied by peritumoral macrophage infiltration and this indicates a poor prognosis [16].

In our study, we did not determine a relationship between NLR and the presence or size of the nodules. This suggests that an increased NLR had no effect on benign pathologies. Among our patients with a nodule, three had a malignant transformation. Our study group included patients with benign pathologies. In these cases, a malignant transformation may be observed over time due to exposure to stress, smoking, or head-neck radiotherapy, with an increase in NLR.

Various studies have investigated the role of platelet count and structure on inflammation. In the study by Yu et al., comparing 280 patients with thyroid cancer with 280 healthy individuals, they demonstrated lower MPV in malignant cases [17]. On the other hand, Dincel et al. demonstrated no correlation between MPV, platelet count, and nodule histopathogenesis; they also demonstrated that the platelet distribution width (PDW) was lower in papillary thyroid cancer as compared to the benign group [18]. In our study, we determined no difference between platelet count or MPV and the presence or size of nodules in 216 patients. Further studies are required.

Our study has some limitations. The first limitation is the lack of a comparison between NLR values and lymphocyte and neutrophil infiltration within the thyroid tissue and nodules. We believe that studies that would include this comparison will be more valuable. The second limitation is the lack of a long-term follow-up of patients with regard to NLR values and nodule sizes. Furthermore, we excluded patients with chronic diseases, to eliminate the possible effect on NLR values and, thus, our sample size was limited.

## Conclusions

Although there are studies suggesting a relationship between NLR, MPV, and the presence of malignancy and mortality, we determined no such relationship between nodule formation and size in thyroid tissue. We believe that increased NLR is instead related to malignant transformation.
